# Personality as a predictor of complex problem-solving in cross-cultural adaptation: a study of African international students in China

**DOI:** 10.3389/fpsyg.2026.1760855

**Published:** 2026-03-25

**Authors:** Weihua Tan, Cheng Liao, Serge Lee, Yushan Zhang

**Affiliations:** 1 Department of Sociology, College of Public Administration, Hunan Normal University, Changsha, China; 2 School of Sociology, Huazhong University of Science and Technology, Wuhan, China; 3 Division of Social Work, College of Health and Human Services, California State University Sacramento, Sacramento, CA, United States

**Keywords:** African international students, complex problem-solving, cross-cultural adaptation, extroversion, mixed-methods research, personality

## Abstract

**Introduction:**

Cross-cultural adaptation is a multidimensional process shaped by both contextual resources and individual dispositions, yet it remains unclear whether personality operates uniformly across adaptation domains, particularly in relational host contexts such as China. This study examined whether personality tendencies are associated with cross-cultural adaptation among African international students in China, and whether associations vary across holistic, cultural, life, and interpersonal domains.

**Methods:**

We used a cross-sectional, quantitative-dominant design. Survey data were collected from 199 African international students enrolled across seven universities in Changsha, China. Personality tendencies were assessed using the 21-item Extraversion scale from the Eysenck Personality Questionnaire (EPQ). Cross-cultural adaptation was measured with a 39-item Likert-type instrument capturing holistic, cultural, life, and interpersonal adaptation. Analyses included chi-square tests and one-way ANOVA for bivariate comparisons, and OLS regression models with robust standard errors comparing Model 1 (personality only) versus Model 2 (personality + demographic/study-related covariates, with university and major fixed effects). Supplementary semi-structured interviews (*n* = 7) were used to contextualize quantitative patterns.

**Results:**

Extroversion was positively associated with adaptation outcomes, with the most consistent and pronounced pattern observed for interpersonal adaptation. In adjusted models, associations with holistic and life adaptation remained robust, whereas the association with cultural adaptation attenuated after covariate adjustment. Descriptively, 57.7% of extroverted students reported good interpersonal adaptation compared with 9.1% of introverted students. Interview narratives highlighted social communication demands, relationship-building, and culturally embedded interaction norms as salient challenges, providing contextual support for the domain-specific quantitative pattern.

**Conclusion:**

Personality tendencies—especially extroversion—are linked to cross-cultural adaptation in a domain-differentiated manner among African international students in China, with strongest implications for interpersonal adjustment. Findings support the value of differentiated, personality-sensitive student support strategies in international higher education.

## Introduction

In an era of accelerating globalization, the number of international students pursuing higher education abroad has grown substantially, bringing the challenges of cross-cultural adaptation to the forefront of psychological inquiry (Ward and Geeraert, 2016). The successful adaptation of sojourners is not merely a practical concern but a complex psychological process, influenced by a confluence of situational and individual factors (Ward et al., 2001). Among these, personality has long been theorized as a key dispositional trait that shapes how individuals perceive, interact with, and adjust to new cultural environments (Gardner, 1962; Wilson et al., 2013).

Despite a well-established body of research linking broad personality traits—most notably extroversion—to general indicators of cross-cultural adjustment, important conceptual gaps remain. Existing studies have largely treated adaptation as a static outcome, rather than as an ongoing process that requires individuals to navigate uncertainty, interdependent social norms, and culturally embedded expectations. As a result, limited attention has been paid to how basic personality tendencies shape individuals’ capacity to cope with cross-cultural adaptation as a dynamic, real-world problem. Recent clinical research in psychological research provide a useful entry point for addressing that complex problem-solving ability—defined as the capacity to manage ill-structured, dynamic, and socially embedded challenges—is significantly impaired among individuals with disordered personality functioning (Kipman et al., 2022). These findings suggest that personality is closely tied to how individuals confront complex life situations that demand continuous adjustment and strategic decision-making. Building on this insight, the present study does not conceptualize complex problem-solving as a directly measured outcome, but rather employs it as an analytic lens through which cross-cultural adaptation is understood. From this perspective, adapting to life in a new cultural environment can be seen as a form of real-world complex problem-solving, in which individuals must learn, revise, and negotiate behavioral strategies over time.

The case of African international students in China offers a particularly compelling context in which to examine these dynamics. Driven by expanding Sino–African cooperation and increased scholarship opportunities, the population of African students in Chinese universities has grown rapidly over the past two decades (Jia and Ning, 2019). However, adapting to Chinese society poses distinct challenges. China is widely characterized as a collectivistic and relationally oriented society, where social interaction, interpersonal sensitivity, and the cultivation of guanxi (interpersonal connections) play a central role in everyday life (Hwang, 1987). Within such a context, personality traits related to sociability and outward engagement may carry particular adaptive value, especially in domains involving interpersonal communication and social integration.

At the same time, prior research on African students in China has primarily focused on structural factors—such as language barriers, institutional support, and cultural distance—while paying comparatively less attention to how individual personality differences condition adaptation outcomes across distinct domains. This leaves open important questions regarding whether, and in what ways, personality tendencies differentially shape various facets of cross-cultural adaptation.

Therefore, grounded in personality psychology and cross-cultural adaptation research, and informed by the Complex Problem-Solving (CPS) perspective, the present study adopts a quantitative-dominant research design to examine how personality tendencies—specifically extroversion and introversion—are associated with cross-cultural adaptation among African international students in China. Rather than treating adaptation as a unitary construct, this study distinguishes between holistic, cultural, life, and interpersonal adaptation domains, allowing for a more fine-grained assessment of personality effects. By clarifying the role of personality in shaping domain-specific adaptation outcomes within a collectivistic host context, this study aims to contribute to a more nuanced understanding of person–culture interaction and to generate evidence that can inform differentiated support strategies in international higher education.

## Literature review

### Cross-cultural adaptation and acculturation: conceptual foundations and domain structure

Cross-cultural adaptation is commonly defined as the set of psychological, behavioral, and social adjustments individuals make when living in a host cultural environment. Rather than a single end-state, adaptation is a multi-faceted process shaped by continuous interactions between the sojourner and the receiving context. Classic work on student sojourners, including Lysgaard’s (1955) “U-curve” proposition, underscores that adjustment may fluctuate over time as individuals move from initial contact and uncertainty to increasing familiarity and competence.

To theorize why adaptation differs across individuals and contexts, acculturation frameworks provide a foundational lens. Berry’s acculturation model proposes that adaptation strategies (e.g., integration, assimilation, separation, and marginalization) reflect individuals’ orientations toward the host and heritage cultures, as well as constraints and affordances embedded in the host environment. This implies that adaptation outcomes should be understood as person–context products, not solely individual attributes. Recent work further emphasizes that acculturation strategies can shape—or condition—the effects of contextual stressors on adjustment outcomes, underscoring the need to treat adaptation as contingent on how individuals engage with the host context (Hommey et al., 2020). Complementarily, the cross-cultural psychology tradition distinguishes key components of adaptation, such as psychological well-being and sociocultural competence (Landis and Bhagat, 1996; Ward et al., 2001). Taken together, these perspectives justify treating cross-cultural adaptation as domain-specific and support the analytical separation of adaptation into distinct outcome domains.

Consistent with this multi-dimensional view, the present study distinguishes four domains—holistic, cultural, life, and interpersonal adaptation—as outcome domains that capture different demands of the host context (e.g., general functioning, cultural learning, daily living competence, and relational integration). This domain-based approach enables a more precise assessment of whether predictors (including personality) operate uniformly or differentially across aspects of adaptation.

### Established correlates of adaptation: contextual resources and individual capacities

A substantial body of research has identified factors that facilitate or hinder adaptation among international students. At the contextual level, social support networks are repeatedly shown to buffer stress, reduce loneliness, and promote both psychological adjustment and sociocultural functioning (Chen et al., 2003; Mao and Liu, 2016). Social support is not merely an external condition; it is often the mechanism through which students gain cultural information, emotional reassurance, and opportunities for interaction. Language competence constitutes another robust predictor: higher host-language proficiency is associated with lower dissatisfaction and faster adjustment, likely because it improves day-to-day functioning and expands access to social exchange and institutional resources (Church, 1982; Tanaka et al., 1994; Andrade, 2006; Wilczewski and Alon, 2022).

At the individual level, expectations, prior intercultural experience, and culturally appropriate coping strategies have also been linked to adaptation outcomes. For example, Chataway and Berry (1989) found that coping orientations relate to satisfaction and stress among Chinese students in Canada, while Ward et al. (2001) emphasized that individual and situational characteristics jointly shape adjustment. Evidence focused on African students in China similarly highlights that unrealistic expectations may undermine satisfaction and adaptation, whereas social networks, prior cross-cultural experience, and perceived cultural understanding are positively associated with adaptation (Akhtar et al., 2015). Importantly, research on Africans in China also documents the broader sociocultural ecology within which African students adapt—including the presence of African communities, forms of everyday interaction with local institutions and residents, and the opportunities/constraints attached to social embeddedness (Bodomo, 2009). These findings collectively suggest that adaptation requires both resources (support, language, institutional opportunities) and capacities (coping, learning, social engagement), with the relative salience of each varying by domain.

### Personality as a domain-specific predictor under host-culture demands: evidence, mechanisms, and research gap

While contextual resources are crucial, stable individual differences may also systematically shape adaptation—particularly when adaptation depends on initiating interaction, building networks, and sustaining culturally appropriate engagement. Personality has long been linked to intercultural effectiveness; Gardner’s (1962) “international communicator” highlights sociability, empathy, and interpersonal sensitivity as key characteristics. Empirical work further suggests that extroversion is associated with more proactive coping and higher adaptability, and cross-cultural studies indicate that traits such as extroversion and neuroticism predict adjustment outcomes in sojourning contexts (Wilson et al., 2013).

However, two limitations remain in the existing literature. First, much research treats personality–adaptation links as global associations, without testing whether personality predicts different adaptation domains in distinct ways. Theoretically, extroversion should be most consequential for domains requiring frequent social initiative and relational maintenance (e.g., interpersonal adaptation), whereas daily-life adaptation may depend more on language competence and practical learning. Second, the adaptation demands of the host society may condition which personality tendencies become “advantageous.” This is particularly relevant in China, a context often characterized as collectivistic and relationally oriented, where guanxi and social embeddedness are integral to everyday functioning (Hwang, 1987). In such a setting, extroverted tendencies may confer domain-specific advantages by enabling greater interaction, faster acquisition of culturally appropriate norms, and more effective network formation. Consistent with this interactionist logic, recent empirical work in China-linked cross-cultural settings suggests that acculturation-related orientations can shape the association between contextual demands and longer-term outcomes, reinforcing the need to test whether individual dispositions matter more in socially and relationally intensive domains (Agbanyo et al., 2024).

In addition, although cross-cultural adaptation has typically been modeled as adjustment and learning, it can also be interpreted as a form of real-world complex problem-solving: individuals must manage uncertainty, navigate interdependent constraints, revise strategies, and learn from feedback over time. Clinical research showing that personality pathology is associated with deficits in complex problem-solving (Kipman et al., 2022) suggests a theoretically meaningful link between dispositional functioning and the capacity to manage complex, dynamic challenges. Building on this insight, the present study treats complex problem-solving as an analytic lens—not a directly measured outcome—and examines whether basic personality tendencies (extroversion vs. introversion) are associated with domain-specific adaptation outcomes among African international students in China.

### Research objective and questions

Building on prior research showing that cross-cultural adaptation is shaped by both contextual resources and individual dispositions, an important gap remains in understanding whether personality operates uniformly across distinct adaptation domains and whether its role is especially salient in relational host contexts such as China. Addressing this gap, the present study focuses on African international students in China and examines the association between personality tendencies and adaptation outcomes across four domains (holistic, cultural, life, and interpersonal). In doing so, the study aims to refine person–culture interaction accounts and generate evidence relevant to targeted student support in international higher education. To aid interpretation of the quantitative patterns, supplementary semi-structured interviews were used to contextualize how students understood and navigated adaptation challenges in daily academic and social life.

This study employed a cross-sectional survey design to examine whether personality tendencies are associated with cross-cultural adaptation among African international students in Changsha, China. Grounded in personality psychology and cross-cultural adaptation research, the study seeks to clarify (a) whether personality tendencies are related to adaptation outcomes and (b) whether such associations are domain-specific in a collectivistic, relationally oriented host context. Consistent with the revised conceptual framing, “complex problem-solving” is used as an analytic lens to interpret adaptation as a dynamic, real-world task rather than as a directly measured construct. Accordingly, we asked:

*RQ1*: Are personality tendencies (extroversion–ambiversion–introversion) associated with overall (holistic) cross-cultural adaptation among African international students in China?

*RQ2*: Do these associations differ across domains, specifically cultural adaptation, life adaptation, and interpersonal adaptation?

*RQ3*: Do the main associations remain after accounting for relevant covariates?

## Methods

### Study design and participants

This study employed a cross-sectional, quantitative-dominant design to examine associations between personality tendencies and cross-cultural adaptation among African international students in Changsha, China. Consistent with the revised manuscript framing, the study tests correlational associations rather than causal effects, and it uses a domain-specific adaptation framework (holistic, cultural, life, and interpersonal) as the primary outcome structure. Although semi-structured interviews were conducted, they served a supplementary, interpretive purpose to contextualize survey patterns rather than constituting a convergent mixed-methods design with parallel qualitative inference. Specifically, interview materials were used to (a) illustrate how participants described key adaptation difficulties and (b) provide contextual interpretation of the quantitative domain patterns; they were not used to generate standalone qualitative claims or to conduct a parallel inferential stream. Ethical approval was obtained from the Ministry of Education of China (Approval #: S201910542026), and all procedures complied with the ethical standards of Hunan Normal University. Participants were recruited from all seven universities in Changsha authorized to enroll African students, as confirmed by the Hunan Provincial Bureau of Education (Central South University, Hunan University, Hunan Normal University, Changsha University of Science & Technology, Hunan University of Technology and Business, Hunan University of Chinese Medicine, and Changsha Medical University). Including all eligible universities in the study setting enhances coverage and heterogeneity within Changsha; however, the sample should be interpreted as context-specific rather than nationally representative of African students in China.

Survey recruitment followed a proportional-convenience approach coordinated through the International Cooperation and Exchange (ICE) offices at each university, which were asked to facilitate participation approximating 12–17% of their African student body. Between November 1 and December 31, 2019, 250 survey packets were distributed and 238 were returned (response rate = 95.2%). After screening for missing data and low-quality responses, 199 questionnaires were retained for analysis (valid rate = 79.6%). The final sample comprised 51.3% men (*n* = 102) and 48.7% women (*n* = 97), with a mean age of 23.33 years (SD = 3.53). Respondents represented 29 African nations, with the largest groups from Nigeria (26.6%), Zimbabwe (15.1%), and Ghana (11.6%), and most participants were undergraduates (83.4%).

Quantitative data were collected using a structured questionnaire that included demographic items (13 nominal indicators), personality tendencies measured by the 21-item Extraversion (E) scale from Eysenck’s Personality Questionnaire (EPQ; Eysenck and Eysenck, 1975; Cronbach’s *α* = 0.719), family factors (13 nominal/ordinal items), and cross-cultural adaptation assessed by a 39-item Likert-type scale covering holistic, cultural, life, and interpersonal domains (Cronbach’s *α* = 0.852). The instrument was professionally translated into Chinese, and the English version was edited by native English-speaking scholars at Hunan Normal University to ensure linguistic and conceptual clarity. To support interpretation of the survey findings, seven survey respondents from Central South University and Hunan Normal University participated in semi-structured interviews focusing on perceived adaptation challenges, coping experiences, and social support; interviews were conducted in private settings, audio-recorded with prior consent, and transcribed verbatim. Following common “supplementary qualitative” practice, excerpts were incorporated selectively to anchor interpretation of the survey results (e.g., interpersonal communication barriers and relational navigation), and the full qualitative materials were documented as supplementary content.

### Measures

#### Dependent variable: cross-cultural adaptation

Cross-cultural adaptation has been defined as changes in cultural patterns arising from sustained and direct intercultural contact (Redfield et al., 1936). Ward and Kennedy (1992) further proposed that adaptation involves psychological, social, and cultural dimensions, and Ward et al. (2001) advanced the EBC model (emotion–behavior–cognition) to describe key components of adjustment. Because international student adaptation involves both general functioning and domain-specific challenges, this study operationalized adaptation as a multi-domain construct measured by a 39-item Likert-type scale. The scale demonstrated strong internal consistency in this sample (Cronbach’s *α* = 0.852). Using Ward’s EBC model as a guiding framework, adaptation was assessed across four outcome domains: (1) holistic adaptation, reflecting an overall evaluation of adaptation across daily living, cultural learning, and interpersonal functioning; (2) cultural adaptation, reflecting culturally compatible knowledge and skills; (3) life adaptation, reflecting adjustment to everyday living requirements; and (4) interpersonal adaptation, reflecting comfort and competence in social interaction with host-country members.

To evaluate construct validity, the KMO and Bartlett’s test of sphericity were conducted (KMO = 0.834; Bartlett’s Sig. < 0.001), indicating suitability for factor analysis. Exploratory factor analysis extracted three core factors (cultural, life, and interpersonal adaptation), and holistic adaptation was retained as a global indicator summarizing respondents’ overall perceived adjustment rather than as a separate factor. Example items include cultural adaptation (“I think I have a good understanding of Chinese after completing my study in China”), life adaptation (“I am proficient in using local public transport”), and interpersonal adaptation (“I really enjoy making new friends with Chinese”). Responses ranged from “strongly disagree” to “strongly agree.”

#### Independent variable: personality tendencies

Personality tendencies were assessed using the Extraversion (introversion–extraversion) scale from the Eysenck Personality Questionnaire (EPQ; Eysenck and Eysenck, 1975; Eysenck et al., 1985). The scale consists of 21 dichotomous (Yes/No) items and demonstrated acceptable internal consistency in the present sample (Cronbach’s *α* = 0.719). For analytic clarity and comparability with prior EPQ-based research, respondents were classified into three groups based on Eysenck’s scoring guidelines: extroversion, ambiversion, and introversion. Specifically, respondents with a total “Yes” score of 15 or above were categorized as extroverted; those scoring 7–14 were classified as ambiverted; and those scoring 6 or below were classified as introverted.

#### Control variable

Covariates were included to adjust for potential confounding in the association between personality tendencies and adaptation outcomes. These variables included gender, age, marital status, enrollment campus, major of study, and length of stay in China. All covariates were selected *a priori* as plausible correlates of exposure and adjustment and were retained in adjusted models regardless of statistical significance. Accordingly, we avoid interpreting non-significant covariate coefficients as evidence of “no effect,” and instead report adjusted associations in the Results.

#### Analytic strategies

Quantitative analyses were performed using SPSS 24.0. Descriptive statistics summarized sample characteristics. Chi-square tests examined bivariate associations between personality categories and adaptation levels. One-way ANOVA compared mean adaptation scores across personality groups for holistic and domain-specific outcomes. To estimate covariate-adjusted associations, multiple regression models were fitted separately for holistic adaptation and each domain-specific outcome, with personality tendency entered as the focal predictor and demographic/study-related variables entered as covariates. To align with common journal presentation, each outcome was estimated using two specifications: Model 1 included personality tendency only, and Model 2 added the full set of covariates (Controls: Yes/No). In addition, MANOVA tested whether personality tendencies had an overall multivariate association across adaptation dimensions as a robustness check. All statistical inferences were interpreted as associations consistent with the cross-sectional design. Qualitative materials were analyzed for supplementary purposes via focused content review anchored to the four adaptation domains (holistic/cultural/life/interpersonal), and excerpts were reported only to illuminate mechanisms suggested by the quantitative patterns rather than to establish independent thematic claims.

## Results

### Personality tendencies of respondents

As measured by Eysenck’s EPQ Extraversion scale, the personality profile of African international students in Changsha was dominated by ambiversion and extroversion (Table 1). Specifically, 58.8% (*n* = 117) were classified as ambivert, followed by 35.7% (*n* = 71) classified as extrovert, whereas only 5.5% (*n* = 11) were classified as introvert. Given the small size of the introvert group (*n* = 11), findings involving this subgroup should be interpreted with appropriate caution, particularly for categorical cross-tabulations.

**Table 1:** Frequency distribution of different personality tendencies (*n* = 199).

Personality tendency	Frequency	Valid percentage (%)	Cumulative percentage (%)
Introvert disposition	11	5.5	5.5
Ambivert	117	58.8	64.3
Extrovert disposition	71	35.7	100

### Cross-cultural adaptation of African students in China

Adaptation scores were categorized into three levels (poor, general, and good) across four domains (Table 2). For holistic adaptation, nearly half of respondents reported good adaptation (48.7%), followed by general (47.2%) and poor (4.0%). For cultural adaptation, general adaptation was most common (52.8%), followed by good (43.2%) and poor (4.0%). For life adaptation, good adaptation accounted for the largest proportion (59.8%), with relatively few reporting poor adaptation (3.0%). By contrast, interpersonal adaptation showed the highest concentration of difficulty, with 13.6% reporting poor interpersonal adaptation, and equal proportions reporting general and good adaptation (43.2% each). Overall, the descriptive pattern suggests generally moderate-to-good adjustment in this Changsha-based sample, with interpersonal adaptation emerging as the most challenging domain.

**Table 2:** Frequency distribution of adaptation of African students (*n* = 199).

Adaptation level	Holistic adaptation		Cultural adaptation		Life adaptation		Interpersonal adaptation	
	Freq.	Val. *P*(%)	Freq.	Val. *P*(%)	Freq.	Val. *P*(%)	Freq.	Val. *P*(%)
Poor adaptation	8	4.0	8	4.0	6	3.0	27	13.6
General adaptation	94	47.2	105	52.8	74	37.2	86	43.2
Good adaptation	97	48.7	86	43.2	119	59.8	86	43.2
Total	199	100.0	199	100.0	199	100.0	199	100.0

### Bivariate associations between personality and adaptation categories (chi-square tests)

To examine whether adaptation categories differed by personality tendency, cross-tabulations and Pearson chi-square tests were conducted (Table 3). For holistic adaptation, extroverted respondents were more likely to report good adaptation (62.0%), whereas introverted respondents predominantly reported general adaptation (81.8%). This association was statistically significant (*χ*^2^ = 24.785, *p* = 0.001).

**Table 3:** Cross analysis of disposition and adaptation (*n* = 199).

Adaptation domain		Poor adaptation		General adaptation		Good adaptation		Pearson’s χ² / *P* value
		Freq.	Pct. (%)	Freq.	Pct. (%)	Freq.	Pct. (%)	
Holistic adaptation	Introverted disposition	0	0.0	9	81.8	2	18.2	Pearson’s chi-square = 24.785 *P* = 0.001
	Ambivert	4	3.4	62	53.0	51	43.6	
	Extroverted disposition	4	5.6	23	32.4	44	62.0	
	total	8	4.0	94	47.2	97	48.7	
Cultural adaptation	Introverted disposition	0	0.0	6	54.5	5	45.5	Pearson’s chi-square = 5.019 *P* = 0.083
	Ambivert	4	3.4	70	59.8	43	36.8	
	Extroverted disposition	4	5.6	29	40.8	38	53.5	
	total	8	4.0	105	52.8	86	43.2	
Life adaptation	Introverted disposition	0	0.0	5	45.5	6	54.5	Pearson’s chi-square = 11.656 *P* = 0.118
	Ambivert	2	1.7	53	45.3	62	53.0	
	Extroverted disposition	4	5.6	16	22.5	51	71.8	
	total	6	3.0	74	37.2	119	59.8	
Interpersonal adaptation	Introverted disposition	6	54.5	4	36.4	1	9.1	Pearson’s chi-square = 25.119 *p* = 0.000
	Ambivert	16	13.7	57	48.7	44	37.6	
	Extroverted disposition	5	7.0	25	35.2	41	57.7	
	total	27	13.6	86	43.2	86	43.2	

For cultural adaptation, the distribution across personality categories was less differentiated, and the association was not statistically significant (*χ*^2^ = 5.019, *p* = 0.083). Similarly, for life adaptation, although extroverted respondents showed a higher proportion of good adaptation (71.8%) than ambiverts (53.0%) and introverts (54.5%), the overall association was not statistically significant (*χ*^2^ = 11.656, *p* = 0.118).

In contrast, interpersonal adaptation exhibited a pronounced pattern by personality tendency (*χ*^2^ = 25.119, *p* < 0.001). More than half of introverted respondents (54.5%) reported poor interpersonal adaptation, compared with 13.7% of ambiverts and 7.0% of extroverts. This domain-specific pattern is consistent with the expectation that sociability-related tendencies are especially consequential for interpersonal adjustment in a relational host context. Supplementary interview excerpts reported below provide contextual illustrations of how limited host-language use and reduced host-national contact can co-occur with interpersonal difficulties, thereby helping interpret this domain pattern without constituting an independent qualitative result stream.

### Mean differences in adaptation scores by personality group (ANOVA)

One-way ANOVA was used to test mean differences in adaptation scores across the three personality groups (Table 4). Results indicated no statistically significant group differences in life adaptation (*F* = 2.130, *p* = 0.121). However, significant mean differences were observed for holistic adaptation (*F* = 6.564, *p* = 0.002), cultural adaptation (*F* = 3.899, *p* = 0.022), and interpersonal adaptation (*F* = 10.922, *p* < 0.001).

**Table 4:** ANOVA table of disposition and adaptation of African students (*n* = 199).

Adaptation domain	95% confidence intervals			*F*	*P*	Group difference comparison
	G1 Introverted disposition (*n* = 11)	G2 Ambivert (*n* = 117)	G3 Extroverted disposition (*n* = 71)			
Holistic adaptation	(26.3933, 35.0613)	(33.7742, 36.9609)	(36.9645, 41.4862)	6.564	0.002	G3 > G2 G3 > G1
Cultural adaptation	(8.3877, 10.8850)	(9.0685, 10.0427)	(9.9998, 11.3242)	3.899	0.022	G3 > G2
Life adaptation	(11.5050, 15.2223)	(13.3806, 14.7049)	(14.0208, 15.8947)	2.130	0.121	/
Interpersonal adaptation	(5.2120, 10.2426)	(10.9797, 12.5588)	(12.6582, 14.5528)	10.922	0.000	G2 > G1 G3 > G1 G3 > G2

Pairwise comparisons (Table 4) showed that extroverted respondents scored higher than ambiverts and introverts on holistic adaptation (G3 > G2; G3 > G1), and higher than ambiverts on cultural adaptation (G3 > G2). For interpersonal adaptation, both ambiverts and extroverts scored higher than introverts (G2 > G1; G3 > G1), and extroverts also exceeded ambiverts (G3 > G2). Taken together, the ANOVA results reinforce a domain-specific conclusion: personality-related differences are most evident for holistic and interpersonal adaptation, whereas life adaptation appears comparatively less sensitive to personality grouping.

### Regression models linking personality tendencies to adaptation outcomes

To complement the categorical comparisons, OLS regression models with robust standard errors were estimated for each adaptation outcome (Table 5). Given the cross-sectional design, results are interpreted as associational rather than causal. Model 1 included personality only, whereas Model 2 added demographic and study-related controls (age, gender, marital status, and length of stay) as well as university and major fixed effects (Controls: Yes/No).

**Table 5:** Regression analysis of personality tendency and adaptation of African students (*n* = 199).

Predictor	Holistic adaptation		Cultural adaptation		Life adaptation		Interpersonal adaptation	
	Model 1	Model 2	Model 1	Model 2	Model 1	Model 2	Model 1	Model 2
Personality (EPQ Extraversion score)	0.728*** (0.163)	0.670*** (0.164)	0.090* (0.042)	0.082^†^ (0.044)	0.154* (0.076)	0.173* (0.079)	0.484*** (0.082)	0.414*** (0.078)
Age	—	0.148 (0.224)	—	−0.019 (0.056)	—	0.084 (0.102)	—	0.083 (0.101)
Male (ref = Female)	—	3.438** (1.202)	—	0.412 (0.323)	—	0.997^†^ (0.557)	—	2.029*** (0.596)
Married/Other (ref = Unmarried)	—	−0.210 (2.168)	—	0.179 (0.582)	—	−0.199 (0.960)	—	−0.190 (1.073)
Length of stay in China (years)	—	0.586 (0.480)	—	0.113 (0.122)	—	0.527** (0.199)	—	−0.054 (0.246)
Controls (demographics + university and major FE)	No	Yes	No	Yes	No	Yes	No	Yes
Constant	22.577*** (2.221)	19.443** (6.173)	5.111*** (0.606)	5.637** (1.700)	12.142*** (1.041)	7.230* (3.175)	5.324*** (1.146)	6.577* (2.836)
*N*	199	199	199	199	199	199	199	199
*R* ^2^	0.095	0.233	0.024	0.088	0.021	0.188	0.153	0.336
Adj. *R*^2^	0.090	0.161	0.019	0.002	0.017	0.112	0.148	0.273

Unstandardized coefficients are reported, with robust standard errors in parentheses. Model 1 includes personality tendency only. Model 2 additionally adjusts for age, gender, marital status, length of stay in China, and university and major fixed effects. ^†^p < 0.10, *p < 0.05, **p < 0.01, ***p < 0.001.

Across outcomes, Model 1 indicates that stronger extroversion tendencies are positively associated with holistic, cultural, life, and interpersonal adaptation. After adding controls in Model 2, the positive association remains robust for holistic, life, and interpersonal adaptation, while the association with cultural adaptation is attenuated and becomes comparatively weaker (marginal by conventional thresholds). Overall, the pattern is consistent with the domain-specific interpretation: personality is most strongly and consistently linked to interpersonal adaptation, converging with the chi-square and ANOVA results, and suggesting that sociability-related tendencies are particularly relevant for social and relational adjustment in the host context. The supplementary interview excerpts below provide contextual illustrations of how language use and host-national contact relate to perceived interpersonal difficulties, without constituting an independent qualitative results stream.

### Supplementary qualitative insights (contextualizing the interpersonal domain)

Seven respondents participated in semi-structured interviews, and all seven transcripts were used selectively as supplementary material. Supplementary excerpts were used illustratively to contextualize the domain-specific quantitative pattern—particularly the prominence of interpersonal difficulties. Several participants linked smoother day-to-day interaction to host-language use (e.g., “Life in China is interesting—especially if you can speak Chinese … many people you can interact with,” Participant 1). Conversely, some participants described limited Chinese proficiency and reduced contact with host nationals alongside interpersonal challenges (e.g., “I do not speak Chinese … I have no Chinese friends and I’m not good at socializing,” Participant 2). Others noted that having even a small number of Chinese friends could facilitate everyday navigation and access to informal support (e.g., “Yes, I have some Chinese friends,” Participant 3). Consistent with the supplementary intent, these excerpts are not presented as systematically derived themes or as representative prevalence claims; rather, they provide contextual illustrations that help interpret why interpersonal adaptation shows the strongest and most consistent association with extroversion in the quantitative models.

## Discussion

### Key findings

This study examined whether personality tendencies are associated with cross-cultural adaptation among African international students in China and whether these associations vary across adaptation domains. Consistent with the study’s aims, extroversion tendency was positively associated with cross-cultural adaptation overall, with the most consistent and pronounced pattern observed for interpersonal adaptation. Importantly, this pattern held not only in bivariate comparisons (chi-square/ANOVA) but also in regression models that contrasted Model 1 (personality only) with Model 2 (personality + controls). This domain-sensitive pattern is consistent with classic cross-cultural transition research showing that stable dispositions are more predictive of “social functioning” facets of adjustment than of domains driven primarily by situational learning demands (Searle and Ward, 1990; Ward and Kennedy, 1999).

Across the four domains, the regression results suggest a clear domain-specific structure: the association between personality and interpersonal adaptation remained robust after adjusting for demographic and study-related characteristics, and the association with holistic adaptation also remained stable. By contrast, the association with cultural adaptation was weaker once controls were included (i.e., attenuated to a marginal level), indicating that cultural adaptation may be more sensitive to contextual or background factors than to personality alone. Life adaptation showed a smaller but still positive relationship with personality, suggesting that dispositional factors may matter even for practical adjustment, though less strongly than for interpersonal functioning. One implication is measurement-concept alignment: interpersonal adaptation in this study closely tracks the broader construct of sociocultural adaptation—often operationalized as interactional competence and “fitting in”—where extroversion-related tendencies plausibly exert their strongest leverage (Ward and Kennedy, 1999).

The observed domain pattern aligns with mainstream acculturation perspectives that distinguish between *what* individuals need to adapt to and *how* adaptation is achieved. Drawing on Ward’s framework (e.g., culture-learning and stress–coping traditions), interpersonal adaptation is inherently embedded in social interaction and relational competence; as a result, it is plausibly more contingent on sociability-related traits. In a host context often described as relationally oriented, where everyday navigation may depend on building and maintaining social ties (e.g., *guanxi*), extroversion may function as a practical interpersonal resource that facilitates contact initiation, network expansion, and repeated interaction—mechanisms directly relevant to interpersonal adaptation. Empirically, research on international students’ friendship networks shows that greater social connectedness and network embeddedness are linked to better affective and evaluative outcomes (e.g., lower homesickness, higher satisfaction), supporting a plausible pathway from sociability-related tendencies to stronger interpersonal adjustment (Hendrickson et al., 2011). Moreover, because interaction opportunities increasingly occur through mediated channels, online social ties can also contribute to cross-cultural adaptation (Rui and Wang, 2015), suggesting that “relational capacity” may be enacted both offline and online in ways that are compatible with extroversion advantages.

At the same time, the weaker (attenuated) association for cultural adaptation in Model 2 suggests that cultural adaptation may depend more heavily on factors such as prior exposure, language-mediated cultural learning, institutional context, and duration/quality of contact—variables that are only partially captured by a single personality tendency measure. This distinction helps reconcile why personality can be strongly predictive in some adaptation domains (interpersonal) while less decisive in others (cultural), even within the same population. Relatedly, recent measurement critiques caution that “adaptation,” “cultural distance,” and “acculturation orientation” are conceptually adjacent but empirically separable; as a result, correlates (including personality) may shift depending on how cultural adaptation is operationalized (Demes and Geeraert, 2014).

However, complex problem-solving is best treated here as an analytic lens for understanding cross-cultural adaptation as an ongoing task marked by uncertainty, shifting constraints, and iterative strategy adjustment. From this perspective, adaptation unfolds through repeated cycles of interpreting culturally embedded cues and norms, selecting and enacting behavioral responses, monitoring social feedback, and updating future strategies accordingly. Within such a process, extroversion may confer an advantage especially in the socially interdependent components of adaptation, where progress hinges on initiating contact, sustaining interaction, and learning from relational feedback. This mechanism offers a plausible explanation for why extroversion is most consistently linked to interpersonal adaptation, and why improvements in the interpersonal domain may, in turn, contribute indirectly to broader (holistic) adjustment. Finally, communication scholarship further suggests that cross-cultural adaptation is partly realized through evolving interaction routines and communication patterns; insofar as extroversion increases the frequency and diversity of communicative encounters, it may accelerate such interactional calibration processes (Peng and Wu, 2019).

### Implications for support in international higher education

The findings suggest that student support services may benefit from moving beyond one-size-fits-all programming toward differentiated, personality-sensitive supports. For example, orientation and ongoing programming can provide multiple pathways to integration: larger social events that suit highly sociable students, and smaller structured activities (e.g., interest-based groups, mentorship pairings, and task-focused workshops) that reduce interaction costs for more introverted students. Advising and counseling services may also usefully incorporate discussions of social comfort and coping styles, helping students select adaptive strategies aligned with their dispositional tendencies while still encouraging gradual, supported engagement with host-country social environments.

### Theoretical contribution

This study contributes to personality and cross-cultural psychology in three ways. First, it shifts from treating cross-cultural adaptation as a unitary outcome to a domain-differentiated construct (holistic, cultural, life, interpersonal), enabling a more precise account of *where* personality matters most. Second, the findings support a person–situation interactionist interpretation: the functional value of extroversion is not assumed to be universal but appears especially consequential in domains where the host environment places high demands on social engagement and relationship-building. Third, by articulating adaptation as a real-world complex task (without reifying CPS as an outcome variable), the study offers a conceptually integrative way to connect acculturation research with broader psychological accounts of adaptive functioning in dynamic environments.

### Limitations and directions for future research

Several limitations should guide interpretation. First, the data are cross-sectional; observed associations should not be interpreted as causal. Second, the study is context-specific to universities in Changsha; while multi-campus coverage increases heterogeneity within this setting, broader generalization to all African students in China requires caution. Third, the small size of the introvert group limits precision when comparing categories. Future studies should employ longitudinal designs to examine adaptation trajectories over time and test whether changes in social networks, coping strategies, and perceived cultural distance mediate personality–adaptation associations. Expanding to multi-province samples and incorporating additional dispositional and contextual variables would further strengthen theory and improve the specificity of practical recommendations.

## Conclusion

This study contributes to cross-cultural adaptation research by demonstrating that personality tendencies—particularly extroversion—are associated with adaptation outcomes among African international students in China, and that these associations vary across adaptation domains. The most consistent pattern concerns interpersonal adjustment, highlighting the relevance of sociability-related traits in a relational host context. By clarifying adaptation as the outcome and positioning complex problem-solving as an interpretive lens, the study strengthens the theoretical account of person–culture interaction and underscores the value of domain-specific modeling of adaptation. Practically, the findings support the development of differentiated student support strategies that accommodate diverse social comfort zones and facilitate multiple routes toward successful integration in international higher education.

## Data Availability

The datasets collected for this study are available on request to the corresponding author.
